# Influence of Hyperglycemia Associated with Enteral Nutrition on Mortality in Patients with Stroke

**DOI:** 10.3390/nu11050996

**Published:** 2019-04-30

**Authors:** Juan José López-Gómez, Esther Delgado-García, Cristina Coto-García, Beatriz Torres-Torres, Emilia Gómez-Hoyos, Cristina Serrano-Valles, Ángeles Castro-Lozano, Juan F. Arenillas-Lara, Daniel A. de Luis-Román

**Affiliations:** 1 Servicio de Endocrinología y Nutrición. Hospital Clínico Universitario Valladolid (HCUV), 47003 Valladolid, Spain; delgadogarciaesther@gmail.com (E.D.-G.); beatriztorrestorres@hotmail.com (B.T.-T.); emiliagomezhoyos@gmail.com (E.G.-H.); CRIS_CALI@hotmail.es (C.S.-V.); acastrolozano@yahoo.es (Á.C.-L.); dadluis@yahoo.es (D.A.d.L.-R.); 2 Centro de Investigación Endocrinología y Nutrición, 47003 Valladolid, Spain; 3 Universidad de Valladolid, 47003 Valladolid, Spain; cristinaz90_151@msn.com; 4 Servicio de Neurología. Hospital Clínico Universitario Valladolid (HCUV), 47003 Valladolid, Spain; juanfarenillas@gmail.com; 5 Instituto de Biología y Genética Molecular (IBGM), 47003 Valladolid, Spain

**Keywords:** hyperglycaemia, stroke, enteral nutrition, nasogastric tube, dysphagia

## Abstract

Objectives: To evaluate in patients admitted for stroke: (1) The frequency of hyperglycaemia associated with enteral nutrition (EN). (2) The risk of morbidity and mortality associated with the development of this type of hyperglycaemia. Methods: A longitudinal observational study was conducted in 115 non-diabetic patients admitted for stroke with EN. Age, functional capacity (Rankin scale), and blood plasma glucose (BPG) were recorded. Hyperglycaemia was considered as: a value higher than 126 mg/dL before the EN and/or a value higher than 150 mg/dL after a week of enteral nutrition. According to this, three groups were differentiated: HyperES: Those who had hyperglycemia before the beginning of the EN (33% patients); NoHyper: those who did not have hyperglycemia before or after (47.8% patients); and HyperEN: Those who did not have hyperglycemia before but suffered it after the beginning of the EN (19.1% patients). Results: The age was 72.72 (15.32) years. A higher rate of mortality was observed in the HyperEN group 45.50%, than HyperES 15.80% or NoHyper: 10.90%). A lower recovery of the oral feeding was observed in those patients of the HyperEN group 27.30%, than HyperES: 42.10% or NoHyper: 61.80%). In the multivariate analysis adjusting for age, sex, and Rankin scale the development of hyperglycemia in those who did not have it at the beginning (HyperEN) was an independent risk factor for non-recovery of the oral feeding (OR: 4.21 (1.20–14.79), *p* = 0.02); and mortality adjusted for age, sex and Rankin scale (OR: 6.83 (1.76–26.47), *p* < 0.01). Conclusions: In non-diabetic patients admitted for stroke with EN, the development of hyperglycaemia in relation to enteral nutrition supposes an independent risk factor for mortality and for the non-recovery of the oral feeding.

## 1. Introduction

Stroke is a neurological disorder derived from alterations in vascularization at the level of the central nervous system (CNS). They are divided into ischemic and haemorrhagic. Stroke is a disorder that affects 15 million people a year globally, with ischemic-type representing around 80%–85% of the cases [1]. In Spain, it is the second cause of global death in the general population, with a mortality rate of 11%, being the main cause of death in women [2]. In addition, it constitutes the first cause of permanent disability in adulthood [3]. Studies show that diabetes mellitus (DM) increases the risk of both types of stroke, even more ischemic stroke [4,5,6]. It is estimated that the risk of suffering an ischemic stroke in people with DM is increased 2–3 times in men and 2–5 times in women, and it also doubles the recurrence risk and is related to worse recoveries [7]. Studies have shown that the risk of ischemic stroke is greater than 8% in patients with diabetes. Among patients with ischemic stroke 28% have pre-diabetes and 45% have overt diabetes [8].

Oropharyngeal dysphagia is a symptom identified among 39%–78% of patients in the acute post-stroke phase [9]. Dysphagia is one of the main causes of post-stroke mortality due to its association with complications such as malnutrition or aspiration pneumonia, which occurs in more than 20% of patients [10]. In the patient with dysphagia, nutritional support is usually required by an enteral route. In the hospitalized patient, the prevalence of diabetes mellitus is 17.8% [11]. Within these patients, the incidence of hyperglycaemia can reach 21.5% [12]. The developing stress hyperglycaemia, ranges from 4%–12% of admitted patients and may reach up to 30% in patients with enteral nutrition [13]. In patients with complete enteral nutrition, hyperglycaemia has been reported in 30%–47% of patients, half of them were not diagnosed with diabetes [14].

The development of hyperglycaemia can be related to a worse evolution of stroke-related damage in stroke patients [15]. In patients with previous diagnosed diabetes, a situation of hyperglycaemia produces an alteration in the recanalization associated with a reduced reperfusion, and a direct damage in the tissues [16]. This condition lead to a worse evolution of the zone of ischemic penumbra and increases the morbidity and mortality. This alteration has shown an increase in mortality and a worse functional recovery in patients with ischemic [17,18] or haemorrhagic stroke [19].

In summary, patients with stroke have a high need for complete enteral nutrition through a nasogastric tube. These patients have a high incidence of hyperglycaemia that can lead to a worse evolution of stroke. The aims of this study were to evaluate the frequency of hyperglycaemia associated with enteral nutrition in patients admitted for stroke with enteral nutritional support, and the risk of development of morbidity and mortality associated with hyperglycaemia.

## 2. Methods

### 2.1. Study Design

A retrospective longitudinal observational study was conducted in 115 non-diabetic patients admitted with a diagnostic of stroke and nutritional support through enteral nutrition tube. The study was conducted in patients treated from January 2014 to December 2016. The study was carried out in patients of the health area of ​​Valladolid Este. These patients were referred from the Neurology Service to the Endocrinology and Nutrition Service of the Valladolid Clinical University Hospital (HCUVA) for total enteral nutritional support during admission.

All the patients started enteral nutritional support by nasogastric tube in the first 48 h after the stroke with a normocaloric normoproteic without fibre formula with the following characteristics: 1 kcal/mL; proteins: 16% from total caloric value (TCV); carbohydrates: 54%TCV; fats: 30%TCV. 

The study was evaluated and approved by the Ethics Committee of the Valladolid Clinical University Hospital (HCUVA) with code PI 16–489. This study was conducted according to the guidelines laid down in the Declaration of Helsinki. 

### 2.2. Population of the Study

The population of the study was non-diabetic patients admitted to the Neurology Service for ischemic or haemorrhagic stroke who cannot reach their energy requirements by an oral route. The inclusion criteria were patients with complete enteral artificial nutrition support by nasoenteric or gastrostomy; start of enteral nutrition during the first 72 h of hospital admission; and patients older than 18 years. The exclusion criteria were patients from the Intensive Care Unit; presence of a previous neurodegenerative disease; presence of infectious pathology that is considered as the aetiology of neurological symptoms and can increase metabolic stress; treatment with medications which can interfere in glucose metabolism (glucocorticoids); patients with diabetes mellitus not diagnosed with HbA1c values at the time of admission equal to or greater than 6.5%; and pregnant patients at the time of admission. 

### 2.3. Hypothesis of the Study

The development of enteral nutrition related hyperglycaemia in patients with stroke has a higher rate of complications, length of stay, and death during admission. 

### 2.4. Study Variables

#### 2.4.1. Characteristics of the Patients

The parameters recorded were age (years, measured at the admission), sex, and type of stroke (ischemic or haemorrhagic).

The Rankin scale was used to evaluate the functional capacity. This scale is a method of measuring the capacity before and after the stratified stroke in six points (0: asymptomatic, 1: non-significant disability despite symptoms, 2: mild disability, 3: moderate disability, 4: moderate-severe disability; 5: severe disability, 6: death). Adequate functional capacity was considered a score of 3 or less [20].

#### 2.4.2. Parameters of Carbohydrate Metabolism and Study Groups

Blood plasma glucose was analysed (chemiluminescence immunoassay using a Roche Diagnostics autoanalyser. (Basel, Switzerland)). The measurement was made before the start of enteral nutrition and one week after the start of the same.

Hyperglycaemia was considered: a value greater than 126 mg/dL before enteral nutrition and/or a value higher than 150 mg/dL after one week of enteral nutrition [21].

Patients with diabetes mellitus was excluded by the patient’s clinical history. We also excluded those patients with glycated haemoglobin at admission greater than 6.5% for diabetes and greater than 5.8% for prediabetes.

#### 2.4.3. Non-Diabetic Patients Were Divided into Three Study Groups

- ***Hyperglycaemia associated with enteral nutrition (HyperEN):*** Those who did not have hyperglycaemia before but suffered after the beginning of enteral nutrition.

- ***Stress Hyperglycaemia (HyperEST):*** Those who had hyperglycaemia before the start of enteral nutrition.

- ***No hyperglycaemia (NoHyper):*** Those who did not have hyperglycaemia either before or after the beginning of enteral nutrition.

The Nutrition Unit monitored capillary blood glucose and it was prescribed insulin treatment with the protocol of the hospital. This protocol includes: a sliding scale with insulin Lispro based on capillary glycaemia (values above 150 mg/dL); all patients who present capillary glycaemia above 150 mg/dL started basal insulin (detemir) at a calculated dose of 0.2 IU/kg weight/day divided into two doses (9:00 h and 21:00 h). 

- Variables of evolution

The target variables evaluated were:

- ***Length of stay:*** defined as the difference in days between discharge and admission.

- ***Mortality:*** defined as the death rate of the sample.

- ***Recovery of the oral route:*** Defined as the ability to ingest food orally after stroke with efficacy and safety.

### 2.5. Statistical Analysis

The data were treated using the statistical package SPSS (SPSS for Windows version 15.0, 2008 SPSS INC, Chicago Ill, USA).

Using the calculation based on a normal distribution, a sample with at least 114 individuals should be selected to calculate an estimated proportion of 20% with an accepted error (or precision) of 5% and a confidence level of 95%.

The continuous variables were described as mean (standard deviation (SD)) in case of normal distribution or as median and interquartile range (pp. 25–75) if the distribution was not normal. The qualitative variables were described by absolute and relative frequencies (percentages). The data was collected in tables and represented in the most appropriate graphs for each type of variable.

In the case of quantitative variables, the Kolmogorov–Smirnov test (when the size was greater than 30) was used to determine the normality of the distributions. To study the differences between independent means, the parametric or nonparametric statistical tests required by the application conditions (ANOVA or Kruskal–Wallis) were used. To study the differences between paired variables, Student’s t test for paired variables (normal variables) and Wilcoxon signed-rank tests (non-normal variables) were used. A survival analysis was performed using a log-rank statistic and graphically represented by Kaplan–Meier curves. A multivariate analysis was performed using a binary logistic regression. The level of significance was conventionally set at *p* ≤ 0.05.

## 3. Results

**Figure 1:**
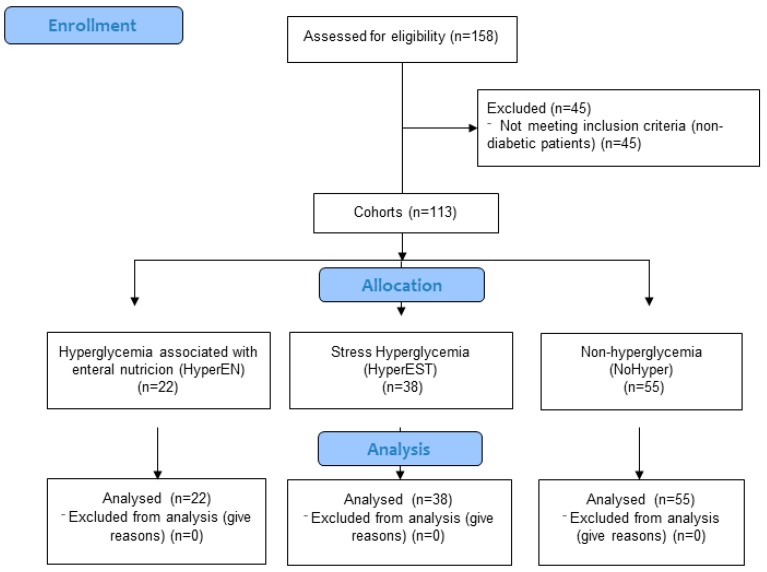
Flow chart of the distribution in cohorts of patients.

A total of 115 stroke patients admitted to the Neurology Service with total enteral nutrition by nasogastric tube in the first 48 h post-stroke were recruited. The distribution in groups of these patients are shown in Figure 1.

### 3.1. Characteristics of Patients

The median age of the sample was 76 (62.5–83) years; with a percentage of men of 63 (54.8%). Of the total of patients 88 (76.5%) suffered ischemic stroke and 27 (23.5%) suffered haemorrhagic stroke. The score of the mean Rankin scale was 0.88 (0–1) with a distribution according to the score: 0 = 63 (54.8%); 1 = 17 (14.8%); 2 = 8 (7%); 3 = 10 (8.7%); 4 = 6 (5.3%); 5 = 4 (3.5%). The length of stay of the patients was 24.34 (11–30.50) days.

#### Characteristics of Carbohydrate Metabolism

Mean blood plasma glycaemia prior to the start of enteral nutrition was 119.73 (29.36) mg/dL and plasma glucose one week after initiation was 133.64 (41.46) mg/dL. Nineteen percent of the patients developed hyperglycaemia associated with enteral nutrition (HyperEN). They developed hyperglycaemia before the start of enteral nutrition before 33% (HyperEST) and 48% did not develop hyperglycaemia either before or after (NoHyper). The differences of the different variables according to the cohort analyzed (HyperEN–HyperEST–NoHyper) are shown in Table 1.

Influence of hyperglycaemia associated with enteral nutrition on the events. The target variables studied were death, recovery of the oral route, and the length of stay.

- Frequencies

**Figure 2:**
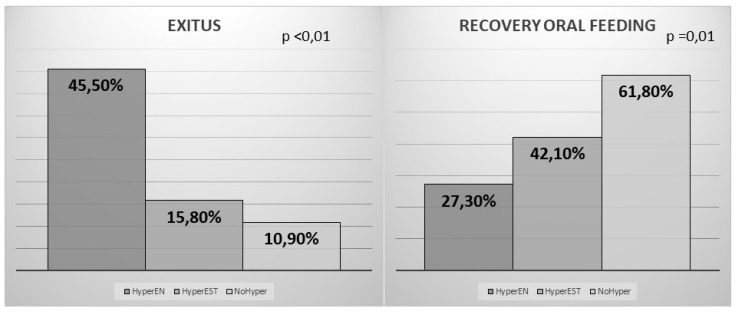
Differences in the percentage of death and patients who recover the oral feeding in the group of hyperglycaemias associated with enteral nutrition (HyperEN), the group that developed hyperglycaemia of stress (HyperEST), and the one that did not develop hyperglycaemia (NoHyper). The differences in the Rankin scale according to the development of hyperglycaemia are shown in Figure 3.

In this study, 22 (19.1%) patients died. The differences regarding on the development or not of hyperglycemia associated with enteral nutrition are shown in Figure 2.

**Figure 4:**
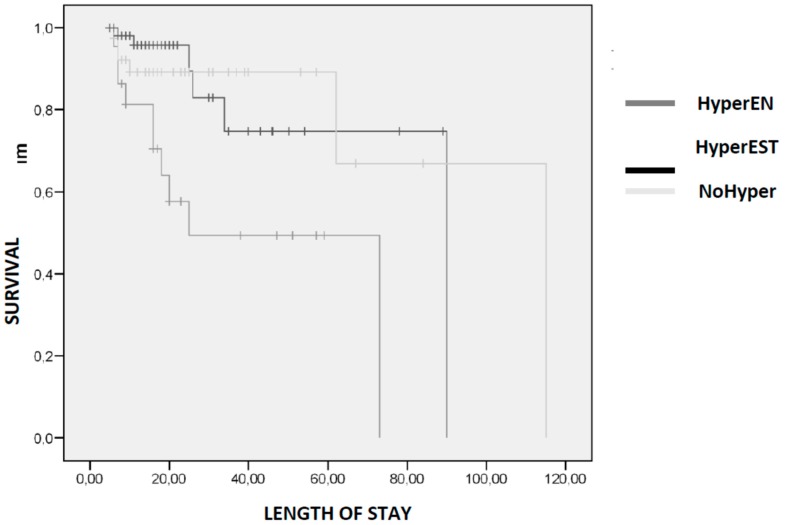
Kaplan–Meier curves comparing survival during admission included patients who developed hyperglycaemia associated with enteral nutrition (HyperEN), those who developed hyperglycaemia of stress (HyperEST), and those who did not develop hyperglycaemia (NoHyper).

A survival analysis was performed comparing those patients who developed hyperglycaemia associated with enteral nutrition (HyperEN), those who developed hyperglycaemia of stress (HyperEST), and those who did not (NoHyper). In patients who died, a greater survival was observed in NoHyper group (25.50 (10.00–48.00) days) with respect to those of the HyperNE group (16 (7–21.25) days and the HyperEST group 8.5 (6.5–75.25) days (*p* < 0.01). The associated Kaplan–Meier curves are shown in Figure 4.

In our sample, 56 (48.7%) recovered oral feeding. The differences according to the development of hyperglycaemia associated with the enteral nutrition are shown in Figure 1.

### 3.2. Multivariate Analysis

For the assessment of the influence of hyperglycaemia associated with enteral nutrition (HyperEN) to death, a multivariate analysis was performed according to the type of stroke (ischemic or haemorrhagic), age, and Rankin scale. An independent relationship was observed between hyperglycaemia and stroke with an odds ratio of 6.83 (1.76–26.47) (Table 2). The probability of death was also analysed when comparing patients who suffered hyperglycaemia from stress without observing significant differences (Table 3).

The relationship between non-recovery of the oral feeding and hyperglycaemia associated with enteral nutrition (HyperEN) was also analysed in relation to the type of stroke, age, and initial Rankin scale. In this case, independent relationship with HyperEN was observed (OR 4.21 (1.20–14.79), *p* = 0.02) (Table 2). In this case, patients with hyperglycaemia of stress (HyperEST) also had a higher probability of not recovering orally (OR 2.68 (1.06–6.72), *p* = 0.04). (Table 3).

## 4. Discussion

Hyperglycaemia is a frequent complication in the patient with complete enteral nutritional support. This study shows that hyperglycaemia associated to complete enteral nutrition increases morbidity and mortality in patients with stroke.

Hyperglycaemia is a frequent situation in hospitalized patient due to different disorders. This complication has been found in up to 21.5% of admitted patients [12]. This complication is difficult to assess in patient with enteral nutrition. Several factors may influence it as the increase in hepatic glucose production and the decrease in peripheral consumption caused by stress and the high levels of cytokines and stress hormones (glucagon, cortisol, and catecholamines). These patients also have an increase in the glycaemic load associated with artificial nutrition [22]. In the patient with enteral nutrition up to 34.5% develop hyperglycaemia [13]. Very few studies differentiate between hyperglycaemia in diabetic and non-diabetic patients. It has been observed that half of patients with hyperglycaemia and enteral nutrition hospitalized do not have a diagnosis of previous diabetes [14,23].

The patient with stroke has an increased tendency to hyperglycaemia as result of a metabolic stress situation. A systematic review of 33 studies conducted by Capes et al. showed hyperglycaemia at admission in the stroke patient of between 8% and 63% [17]. On the other hand, the incidence of initiation of complete enteral nutrition by nasogastric tube in this pathology ranges between 8.5% and 29% [24]. This situation is due to the high rate of dysphagia associated to the pathophysiology of the disease and the recommendation to start enteral nutrition by tube if inadequate intake is expected for more than a week [25]. These two entities (stroke and complete enteral nutrition) are closely related to hyperglycaemia [16].

In this study, it was observed that more than half of the patients suffered some type of hyperglycaemia. One third of the sample suffered hyperglycaemia of stress probably associated with the acute situation related to stroke. The fifth part of the sample suffered hyperglycaemia related to enteral nutrition. This may be related to an increase in glycaemia after the restart of the diet due to the alteration in the carbohydrate metabolism associated with the pathophysiology of the stroke [26,27].

The selection of the study groups was made based on the cause of the hyperglycaemia. The main reason was to differentiate the effects of hyperglycaemia caused by enteral nutrition in non-diabetic patients versus those who had stress hyperglycaemia or who did not develop hyperglycaemia. There are many studies showing the relationship of hyperglycaemia in diabetics and non-diabetics with complications in the patient with enteral nutrition [28], but very few define the hyperglycaemia associated with enteral nutrition [29].

The composition of enteral nutrition may have an important relationship with the development of hyperglycaemia, although the caloric load administered is also important. A study conducted by Valizadeh et al. showed 14.45% of hyperglycaemia in non-diabetics treated with enteral nutrition, a higher rate of hyperglycaemia was observed in those with higher caloric load and a predominance in patients of internal medicine services [29]. In our population, the caloric distribution was similar in all patients and the same type of nutrition was used in all, normoproteic isocaloric without fibre formula.

Patients with hyperglycaemia associated with enteral nutrition were older. This is related to the greater tendency of older people to develop hyperglycaemia defined in studies such as González-Infantino et al. [14]. Likewise, patients with hyperglycaemia associated with enteral nutrition had a worse situation on the Rankin scale before the development of stroke. Although there were no differences when stratifying according to the range of Rankin scale. This difference in the development of hyperglycaemia associated with enteral nutrition in patients with poorer functional capacity is probably related to a more pronounced alteration of the carbohydrate metabolism [30]. In the human body there are different mechanisms by which glycaemic control is maintained and this includes maintaining an optimal balance between insulin and other counterregulatory hormones, like stress-induced hormones and glucagon [31].

Both ischemic and haemorrhagic strokes can be associated with hyperglycaemia in non-diabetics as was observed in studies like INTERACT2 for haemorrhagic [32] or SMART for ischemic [33]. In our study there were no differences between the groups at this level.

A higher baseline glycaemia was observed in the stress hyperglycaemia group and a higher one-week basal glycaemia in the group of hyperglycaemia associated with enteral nutrition. This circumstance probably related to the higher insulin treatment rate in the stress hyperglycaemia group. This situation could be related to the detection of hyperglycaemia at the onset of stroke. It has been observed that glycaemic control by insulin in the patient with hyperglycaemia in stroke, in studies like THIS (Treatment of Hyperglycaemia in Ischemic Stroke) or GRASP (Glucose Regulation in Acute Stroke Patients), produces an improvement in complications and long-term evolution [27,34]. It has been observed also that glycaemic control in the first days after stroke can improve the complication rate [35], although, when an intensive insulin treatment is used, this improvement is not so clear [36].

Hyperglycaemia in stroke patient is associated with a worse evolution. In ischemic strokes it has been observed that a hyperglycaemia maintained during admission is associated with a higher mortality as described by Mi et al. in diabetics (OR 24; 95% CI (2.8–199.3)) and evolution as haemorrhagic transformation (OR: 13.3, 95% CI (2.7–66.1)) [18]. In patients with cerebral haemorrhage, the INTERACT2 study showed that hyperglycaemia and diabetes mellitus are independent predictors with a higher rate of complications and more severe [31]. In the study performed, a higher rate of complications was observed in relation to hyperglycaemia associated with enteral nutrition. It has also been observed that stress hyperglycaemia increases the worse prognosis of patients with stroke in both diabetics and non-diabetics [17]. This condition can be seen in the results of our study. 

On the other hand, the increase in the rate of complications associated with hyperglycaemia diagnosed during support with artificial nutrition is well established, as described by González-Infantino et al. and Valizadeh et al. [14,29]. However, this type of hyperglycaemia has not been analysed in studies in patients with stroke. Hyperglycaemia associated with artificial nutrition is a complication to consider in stroke patients given the high rate of patients who require complete enteral nutrition during admission.

The relationship of hyperglycaemia associated with enteral nutrition with the rate of complications in these patients has not been studied. In our study we showed that patients with a development of hyperglycaemia associated with enteral nutrition independently of stress hyperglycaemia and excluding previous diabetic patients produce an increase in mortality. Sustained hyperglycaemia associated during the seven days after the ischemic stroke has shown an increased risk of mortality (OR: 7.61, 95% CI: 3.23–17.90) as described by Yong et al. [37].

On the other hand, in our study, patients with hyperglycaemia associated with enteral nutrition showed a worse evolution of the stroke measured through the non-recovery of the oral route. Likewise, in a sub analysis of the SMART study, hyperglycaemia was associated with a worse evolution of functional capacity in non-diabetics with ischemic stroke [33]. We must analyse this alteration in functional capacity during admission as we do not have long-term functional recovery data. The existing evidence has not shown in some cases that hyperglycaemia during admission may worsen long-term functional evolution [38].

The main limitation of the study is its retrospective status with the possible loss of data. Another limitation is the difficulty in differentiating the influence of enteral nutrition in the group of patients with hyperglycaemia of stress.

On the other hand, it would be interesting to know the influence of different formulas of enteral nutrition on the control of glycaemia in this type of patients. In our study we used a unique type of formula (normocaloric normoproteic without fibre). Therefore, it would be important to study if the formula change achieves a benefit in variables, such as mortality, recovery of the oral route, or the average stay in patients with stroke.

## 5. Conclusions

In non-diabetic patients admitted for cerebrovascular disease with enteral nutritional support, a fifth suffered hyperglycaemia associated with complete enteral nutrition. Patients with hyperglycaemia associated with enteral nutrition have a higher mortality risk than the others. These patients have a lower recovery of oral feeding than the others.

## Figures and Tables

**Figure 3:**
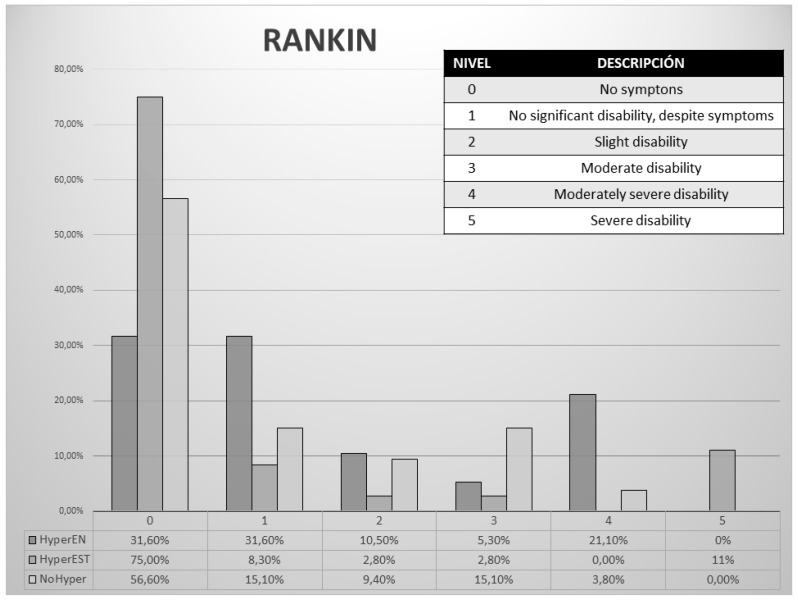
Comparison in functional capacity before stroke according to the Rankin scale depending on the development of hyperglycaemia associated with enteral nutrition (HyperEN), development of hyperglycaemia of stress (HyperEST), or non-development of hyperglycaemia (NoHyper).

**Table 1:** Differences in variables between study groups.

	HyperEN	HyperEST	NoHyper	*p*-Value
***n* (%)**	22 (19.1%)	38 (33%)	55 (47,8%)	
**Sex M/W (%)**	14/8 (63.6%/36.4%)	16/22 (42.1%/57.9%)	33/22 (60%/40%)	0.15
**Ischemic Stroke (%)**	18 (81.8%)	25 (65.8%)	45 (81.8%)	0.16
**Age (years)**	80 (71.75–87)	69.29 (61.50–78.25)	77 (67–85)	0.03
**BMI**	25.13 (4.9)	26.65 (4.86)	25.65 (3.81)	0.39
**HbA1c (%)**	5.6 (0.27)	5.56 (0.27)	5.5 (0.32)	0.59
**BPG before (mg/dL)**	103.64 (13.40)	146.50 (23.99)	101.24 (14.88)	<0.01
**BPG after (mg/dL)**	173.82 (21.99)	152.50 (56.04)	120.62 (18.81)	<0.01
**Rankin**	1 (0–3)	0 (0–0.75)	0 (0–2)	0.03
**Length of stay (days)**	25 (10.91)	27.50 (23.67)	22.16 (19.29)	0.48

(HyperEN: Hyperglycaemia associated enteral nutrition; HyperEST: stress hyperglycaemia; NoHyper: not hyperglycaemia) (OR: Odds Ratio; M: men; W: women; BMI: Body Mass index; HbA1c: glycated haemoglobin; BPG: blood plasma glucose).

**Table 2:** Relationship of hyperglycaemia associated with enteral nutrition (HyperEN) with death and recovery of the oral feeding on the type of stroke, age, and Rankin scale.

**EXITUS**	**OR**	**IC (95%)**	***p*-value**
**HyperEN**	6.83	1.76–26.47	<0.01
**Type of Stroke**	1.46	0.21–10.16	0.70
**Age**	1.04	0.97–1.11	0.26
**Rankin**	1.34	0.80–2.23	0.26
**NOT RECOVERY ORAL FEEDING**	**OR**	**IC (95%)**	***p*-value**
**HyperEN**	4.21	1.20–14.79	0.02
**Type of Stroke**	2.34	0.54–10.06	0.25
**Age**	1.03	0.99–1.08	0.11
**Rankin**	1.41	0.90–2.21	0.13

OR: Odds Ratio.

**Table 3:** Relationship of stress hyperglycaemia (HyperEST) with death according to the type of stroke, age, and ranking.

**EXITUS**	**OR**	**IC (95%)**	***p*-value**
HyperEST	2.18	0.59–8.06	0.24
Type of Stroke	0.62	0.12–3.23	0.57
Age	1.02	0.97–1.08	0.35
Rankin	0.97	0.61–8.06	0.24
**NOT RECOVERY ORAL FEEDING**	**OR**	**IC (95%)**	***p*-value**
HyperEST	2.68	1.06–6.72	0.04
Type of Stroke	0.68	0.23–2.02	0.49
Age	1.01	0.98–1.04	0.58
Rankin	1.36	0.97–1.91	0.08

OR: Odds Ratio.

## References

[1] Ojo O., Brooke J. (2014). Evaluation of the Role of Enteral Nutrition in Managing Patients with Diabetes. Nutrients.

[2] Sanchez Larsen A., García García J., Ayo-Martín O., Hernandez-Fernández F., Diaz Maroto I., Fernández-Díaz E., Monteagudo M., Segura T. (2016). Has the etiology of ischaemic stroke changed in the past decades? Analysis and comparison of data from current and historical stroke databases. Neurología.

[3] Burgos Pelaez R., Segurola Gurrutxaga H., Bretón Lesmes I. (2014). Nutritional Support in stroke patients. Nutr. Hosp..

[4] Tanaka R., Ueno Y., Miyamoto N., Yamashiro K., Tanaka Y. (2013). Impact of diabetes and prediabetes on the short-term prognosis in patients with acute ischemic stroke. J. Neur. Sci..

[5] Hamkey G., Anderson N., Thing R., Veillard A., Romo M., Wosik M. (2013). Rates and predictors of risk of stroke and its subtypes in diabetes: A prospective observational study. J. Neur..

[6] Kagansky N., Levy S., Knobler H. (2001). The role of hyperglycemia in acute stroke. Arch. Neurol..

[7] Mukul Sharma M., Gubitz G. (2013). Management of stroke in diabetes. Can. J. Diabetes.

[8] Vellipuram A.R., Rodríguez G., Rawla P., Maud A., Cruz-Flores S., Khatri R. (2019). Lifestyle interventions to prevent cardiovascular events after stroke and transient ischemic attack. Curr. Cardiol. Rep..

[9] Crary M.A., Humphrey J.L., Carnaby-Mann G., Sambandam R., Miller L., Silliman S. (2013). Dysphagia, nutrition, and hydration in ischemic stroke patients at admission and discharge from acute care. Dysphagia.

[10] Passos K., Cardoso M.C., Scheeren B. (2017). Association between functionality assessment scales and the severity of dysphagia post-stroke. CoDAS.

[11] Carral F., Olveira G., Aguilar M., Ortego J., Gavilán I., Doménech I., Escobar L. (2003). Hospital discharge records under-report the prevalence of diabetes in inpatients. Diabetes Res. Clin. Pract..

[12] Moghissi E.S., Korytkowski M.T., Di Nardo M., Einhorn D., Hellman R., Hirsch I.B., Inzucchi S.E., Ismail-Beigi F., Kirkman M.S., Umpierrez G.E. (2009). American Association of Clinical Endocrinologists and American Diabetes Association consensus statement on inpatient glycemic control. Diabetes Care.

[13] Pancorbo-Hidalgo P.L., García-Fernández F.P., Ramírez-Pérez C. (2001). Complications associated with enteral nutrition by nasogastric tube in an internal medicine unit. J. Clin Nurs..

[14] Gonzalez-Infantino Ca., González C.D., Sánchez R., Presner N. (2013). Hyperglycemia and hipoalbuminemia as prognostic mortality factors in patients with enteral feeding. Nutrition.

[15] Chen R., Ovbiagele B., Feng W. (2016). Diabetes and Stroke: Epidemiology, pathophysiology, pharmaceuticals and outcomes. Am. J. Med. Sci..

[16] Kruyt N.D., Biessels G.J., Devries J.H., Roos Y.B. (2010). Hyperglycemia in acute in ischemic stroke: Pathophysiology and clinical management. Nat. Rev. Neurol..

[17] Capes S.E., Hunt D., Malmberg K., Pathak P., Gerstein H.C. (2001). Stress hyperglycemia and prognosis of stroke in nondiabetic and diabetic patients. Stroke.

[18] Mi D., Wang P., Yang B., Pu Y., Yang Z., Liu L. (2018). Correlation of hyperglycemia with mortality after acute ischemic stroke. Ther. Adv. Neurol. Disord..

[19] Kruyt N.D., Biessels G.J., De Vries J.H., Luitse M.J., Vermeulen M., Rinkel G., Vandertop W.P., Roos Y.B. (2010). Hyperglycemia in aneurysmal subarachnoid hemorrhage: Apotentially modifiable risk factor for poor outcome. J. Cereb. Blood Flow Metab..

[20] Wilson L.J., Harendran A., Grant M., Baird T., Schultz U., Muir K., Bone I. (2002). Improving the assessment of outcomes in stroke: Use of a structured interview to assign grades on the Modified Rankin Scale. Stroke.

[21] McMahon M.M., Nystrom E., Braunschweig C., Miles J., Compher C., American Society for Parenteral and Enteral Nutrition (ASPEN) (2013). Clinical Guidelines: Nutrition Support of adults’ patients with hyperglucemia. JPEN.

[22] Drincic A.T., Knezevich J.T., Akkireddy P. (2017). Nutrition and hyperglycemia management in the inpatient setting (meals on demand, parenteral, or enteral nutrition). Curr. Diab. Rep..

[23] Arinzon Z., Shabat S., Shuval Peisakh A., Berner Y. (2008). Prevalence of diabetes mellitus in elderly patients received enteral nutrition long-term care service. Arch. Gerontol. Geriatr..

[24] Broadley S., Croser D., Cottrell J., Creevy M., Teo E., Yiu D., Pathi R., Taylor J., Thompson P.D. (2003). Predictors of prolonged dysphagia following acute stroke. J. Clin. Neurosci..

[25] Burgos R., Bretón I., Cereda E., Desport J.C., Dziewas R., Genton L. (2018). ESPEN guideline clinical nutrition in neurology. Clin. Nutr..

[26] Bruno A., Saha C., Williams L.S., Shankar R. (2004). IV insulin during acute cerebral infarction in diabetic patients. Neurology.

[27] Bruno A., Kent T.A., Coull B.M., Shankar R.R., Saha C., Becker K.J., Kissela B.M., Williams L.S. (2008). Treatment of hyperglycemia in ischemic stroke (THIS): A randomized pilot trial. Stroke.

[28] Davidson P., Kwiatkowski C.A., Wien M. (2015). Management of hyperglycemia and enteral nutrition in the hospitalized patient. Nutr. Clin. Pract..

[29] Valizadeh M.A., Vahdat Z., Vahabzadeh D., Vahabzadeh Z., Nasiri L., Shargh A. (2017). Non-diabetic hyperglycemia and some of its correlates in ICU hospitalized patients receiving enteral nutrition. Maedica.

[30] Allport L., Baird T., Butcher K., Macgregor L., Prosser J., Colman P., Davis S. (2006). Frequency and temporal profile of poststroke hyperglycemia using continuous glucose monitoring. Diabetes Care.

[31] Rawla P., Vellipuram A.R., Bandary S.S., Pradeep Raj J. (2017). Euglycemic diabetic ketoacidosis: A diagnostic and therapeutic dilemma. Endocrinol. Diabetes Metab. Case. Rep..

[32] Saxena A., Anderson C.S., Wang X., Sato S., Arima H., Chan E., Muñoz-Venturelli P., Delcourt C., Robinson T., Stapf C. (2016). Prognostic significance of hyperglycemia in acute intracerebral hemorrhage. The INTERACT2 Study. Stroke.

[33] Yao M., Ni J., Zhou L., Peng B., Zhu Y., Cui L., SMART investigators (2016). Elevated fasting blood glucose is predictive of poor outcome in non-diabetic stroke patients: A sub-group analysis of SMART. PLoS ONE.

[34] Johnston C.K.C., Hall C.E., Kissela B.M., Bleck T.P., Mark R. (2009). Glucose Regulation in Acute Stroke Patients (GRASP) Trial: A randomized pilot trial. Stroke.

[35] Gentile N.T., Sefchick M.W., Huynh T., Kruus L.K., Gaughan J. (2006). Decreased mortality by normalizing blood glucose after acute ischemic stroke. Acad Emerg. Med..

[36] Gray C.S., Hildreth A.J., Sandercock P.A., O’Connell J.E., Johnston D.E., Cartlidge N.E., Bamford J.M., James O.F., Alberti K.G., GIST Trialists Collaboration (2007). Glucose-potassium-insulin infusions in the management of post-stroke hyperglycaemia: The UK glucose insulin in stroke trial (GIST-UK). Lancet Neurol..

[37] Yong M., Kaste M. (2008). Dynamic of hyperglycemia as a predictor of stroke outcome in the ECASS-II Trial. Stroke.

[38] Ntaios G., Abatzi C., Alexandrou M., Lambrou D., Chatzopoulos S., Egli M., Ru J., Bornstein N., Michel P. (2011). Persisten hyperglycemia at 24–48 h in acute hyperglycemic stroke patients is not associated with a worse functional outcome. Cerebrovasc Dis..

